# Researches into the Anatomy and Pathology of the Eye

**Published:** 1896-12

**Authors:** 


**Researches into the Anatomy and Pathology of the Eye.** By E. Treacher Collins. Pp. xiv., 140. London: H. K Lewis. 1896.

This book is an extension of the lectures given before the Royal College of Surgeons by the author in 1894, and the outcome of six years' work as Curator of the Museum of the Royal London Ophthalmic Hospital.

The pathological researches of Mr. Collins of recent years are not second either in importance or number to those of any other observer, and the book before us does honour to English ophthalmology. It contains the results of an enormous amount of original investigation, and no space is wasted by repetition of knowledge which for some time has been well recognised and fully worked out. The book is well arranged, very clearly written, and has good illustrations. No ophthalmic surgeon should be without it; where points of difficulty are not cleared up, there is always suggestive reasoning for other investigators to elaborate.

The various anomalies of development and diseased conditions of the lens are followed by those which affect all the other tissues of the eyeball. Abnormalities of the iris, and unusual forms of glaucoma, the effects of aneurysm, and of wounds, all are fully considered and largely illustrated. Every page of the book bears evidence of the author's ability and experience, and great credit is due to the publisher for the way the book has been issued.

